# Exploring the differential impact of individual and organizational factors on organizational commitment of physicians and nurses

**DOI:** 10.1186/s12913-018-2977-1

**Published:** 2018-03-15

**Authors:** Felix Miedaner, Ludwig Kuntz, Christian Enke, Bernhard Roth, Anika Nitzsche

**Affiliations:** 1 Department of Business Administration and Health Care Management, University of Cologne, Universitätsstraße 91, 50931 Cologne, Germany; 2 Cologne Center for Ethics, Rights, Economics, and Social Sciences of Health (ceres) and Research Unit Ethics, Medical Faculty, University Clinic Cologne, Cologne, Germany; 3 Department of Neonatology and Paediatric Intensive Care, Children’s Hospital, University of Cologne, Cologne, Germany; 4 Institute of Medical Sociology, Health Services Research, and Rehabilitation Science (IMVR), University of Cologne, Cologne, Germany

**Keywords:** Organizational commitment, Work experiences, Organizational structures, Occupational group, Critical care

## Abstract

**Background:**

Physician and nursing shortages in acute and critical care settings require research on factors which might drive their commitment, an important predictor of absenteeism and turnover. However, the degree to which the commitment of a physician or a nurse is driven by individual or organizational characteristics in hospitals remains unclear. In addition, there is a need for a greater understanding of how antecedent-commitment relationships differ between both occupational groups.

Based on recent findings in the literature and the results of a pilot study, we investigate the degree to which selected individual and organizational characteristics might enhance an employee’s affective commitment working in the field of neonatal intensive care. Moreover, our aim is to examine the different antecedent-commitment relationships across the occupational groups of nurses and physicians.

**Methods:**

Information about individual factors affecting organizational commitment was derived from self-administered staff questionnaires, while additional information about organizational structures was taken from hospital quality reports and a self-administered survey completed by hospital department heads. Overall, 1486 nurses and 540 physicians from 66 Neonatal Intensive Care Units participated in the study. We used multilevel modeling to account for different levels of analysis.

**Results:**

Although organizational characteristics can explain differences in an employee’s commitment, the differences can be largely explained by his or her individual characteristics and work experiences. Regarding occupational differences, individual support by leaders and colleagues was shown to influence organizational commitment more strongly in the physicians’ group. In contrast, the degree of autonomy in the units and perceived quality of care had a larger impact on the nurses’ organizational commitment.

**Conclusions:**

With the growing number of hospitals facing an acute shortage of highly-skilled labor, effective strategies on the individual *and* organizational levels have to be considered to enhance an employee’s commitment to his or her organization. Regarding occupational differences in antecedent-commitment relationships, more specific management actions should be undertaken to correspond to different needs and aspirations of nurses and physicians.

**Trial registration:**

German Clinical Trials Register (DRKS00004589, date of trial registration: 15.05.2013).

## Background

Over the last few decades, a comprehensive body of knowledge has emerged, which addresses the positive influence that an employee’s organizational commitment, i.e., his or her psychological attachment to the organization, can have on an organization’s performance [1–8]. In particular, employees with low commitment to their organization have, e.g., higher turnover [9] or absenteeism [10] rates. In hospitals, there is strong consensus that nurses’ organizational commitment can hamper turnover intentions and stress [11–14], a relationship that is of great importance in times of severe workforce shortages within hospitals, especially in the nurses’, but also the physicians’ occupational group [15]. In addition, a recent study with physicians showed that organizational commitment was found to be positively related to improving patient safety culture [16]. As such, this enhances the need to identify and foster factors that may strengthen employee commitment.

To date, a variety of factors that influence an employee’s commitment to his or her organization have been examined. According to Meyer and Allen [17], these can be broken down into three categories: Demographic characteristics or personal dispositions of an employee (personal characteristics), work-related factors an employee experiences during his or her work (work experiences), and characteristics of the organization in which he or she is working (organizational structures). The latter two can be differentiated by whether the factor influences one particular employee (work experiences) or all employees (organizational structure) within an organization [18]. However, studies examining antecedents of organizational commitment in hospitals tend to focus on only one level of analysis, rather than explaining the effect through variables at both the individual and the organizational level. This could reveal the extent to which an employee’s individual commitment can be explained by each level of analysis. In addition, the research on commitment in healthcare settings tends to focus solely on one particular occupational group, namely nurses, rather than considering all participating professionals who provide healthcare. Surprisingly little has been done to investigate the differential effect of antecedents on organizational commitment for employees from different professions, even though Cohen [19] emphasized early on the fundamental disparities of antecedent-commitment relationships by comparing occupational groups with low and high status.

Using hierarchical multilevel modeling, our study aims to examine factors that influence an employee’s commitment to his or her hospital by differing between individual-level and organizational-level predictors. Results of a pilot study, conducted in five Neonatal Intensive Care Units (NICUs) and including 198 nurses and 70 physicians [20], and recent studies that examine potential drivers of affective commitment formed the basis for the examined antecedents in our study. Moreover, we strive to examine the influence and potentially different effects of these antecedents with regard to the occupational groups of nurses and physicians. The results are intended to provide insights into what management actions should be taken for different occupational groups in order to increase employee commitment.

In the conceptual background of this paper, we first explain the rationale of using affective organizational commitment as a dependent variable; we subsequently draw upon existing research on antecedents as potential drivers of commitment. Therefore, we make use of the basic differentiation in literature between work experiences at individual and organizational structures at organizational level [17, 18]. Finally, we describe the rationale why these associations might differ across the occupational groups of physicians and nurses.

### Organizational commitment

An employee’s attachment towards his or her organization has been studied recently from a number of different perspectives, including the involvement in, commitment to, or engagement with the organization. Although these concepts share the commonality of focusing on a positive bond between employees and their organization, they reflect different aspects of work attachment [21]. While organizational commitment relates to the psychological attachment of a person to the organization, work engagement additionally includes the component of an employee’s physical health [21]. As such, commitment to the organization might be an important prerequisite as to why an employee becomes engaged [22].

As a further development of the basic differentiation between attitudinal and behavioral commitment [9], commitment to an organization is distinguished by three components: normative, continuance, and affective commitment [17]. Normative commitment is based on an employee’s feeling of obligation to remain with an organization, while continuance commitment refers to an employee’s cost-benefit consideration for staying with or leaving an organization. Affective commitment emphasizes the emotional aspect of an employee’s commitment to his or her organization and is defined as “the employee’s emotional attachment to, identification with, and involvement in the organization” [17].

In this study, we focus on the component of affective commitment of physicians and nurses for several reasons. First, since affective commitment measures employee commitment to an organization after an individual has begun working with the organization [23], the degree of commitment depends strongly on what happens in the organization and whether the employee’s individual needs are satisfied by his or her work experiences and underlying organizational structures. Second, in a meta-analysis, Meyer et al. [4] showed that affective commitment has the strongest correlation with employee (stress and work-family conflict) and organizational (attendance, performance, and organizational citizenship behavior) outcomes, compared to the two other forms of commitment. In addition, a direct relationship between higher affective commitment and patient safety as perceived by physicians was found in the hospital setting [16].

### Work experiences

From the individual perspective, various work experiences in an organization have been recently identified for explaining organizational commitment. In a meta-analysis, Humphrey et al. [24] emphasize the importance of social support and autonomy as important antecedents for organizational commitment: The more autonomy a person has in his or her profession, and the more he or she is supported by a team and leader, the higher is an individual’s organizational commitment. As such, we hypothesize the following:

- Hypothesis 1a: *Work experiences (individual autonomy and support from leader and colleagues) are positively associated with an employee’s organizational commitment.*

### Organizational structures

From the organizational perspective, the perceived organizational support and the degree of autonomy within an organization have especially been shown to be predictive characteristics of an individual who is more committed to his or her organization in non-hospital [25, 26] as well as hospital settings [27]. In addition, studies have shown that the perceived quality of nursing practice and nurse-physician collaboration directly influences the commitment of nurses in intensive care [28, 29]. Thus, the perceived quality of care, as well as organizational structures fostering interprofessional collaboration and communication (inter alia, through regular interdisciplinary conferences), could enhance an individual’s commitment to the organization. In addition, following previous findings in a non-hospital setting [26], we argue that commitment in smaller hospitals may be higher because employees can identify more easily with the organization. This leads us to the following hypothesis:

- Hypothesis 1b: *Organizational structures (organizational support from leader and colleagues, organizational autonomy, perceived quality of care, open communication, existence of regular interdisciplinary medical conferences, and smaller hospitals) are positively associated with an employee’s commitment to his or her organization.*

### Differences between occupational groups

Because employees from different professional backgrounds may have different relationships with the organization, factors influencing commitment to the organization could depend on an employee’s membership in a particular occupational group.

Early conceptual models have tried to explain why disparities between the commitment levels of professionals might exist. Drawing upon reference group theory, Gouldner distinguished between two types of professionals: *cosmopolitans*, who are “(…) low on loyalty to the employing organization, high on commitment to specialized role skills”, and *locals,* who display higher levels of loyalty to the organization and who are less committed to specialized role skills [30, 31]. Grusky [32], e.g., found that people who have overcome greater obstacles in comparison to others to receive rewards from the organization typically show greater commitment. Focusing on differences across occupational groups, Cohen [19] argued in a meta-analysis that antecedents of organizational commitment differ across occupational groups with different hierarchical status. More specifically, he found that, to some extent, personal characteristics have a greater impact on commitment for employees in low-status occupations, while structural and work experience antecedents have a greater impact on the employees’ commitment in high-status occupations.

Surprisingly little has been done to investigate potential disparities within hospitals, although substantial differences between the occupational groups of physicians and nurses do exist [33]. In hospitals, physicians usually have the authority to issue directives to nurses on medical issues and make the ultimate decisions about medical treatment. This might lead to differences in status, authority and responsibilities between both occupational groups, which has already been highlighted within the context of intensive care [33, 34]. Following this, the influence of the different factors driving an employee’s commitment might differ between the occupational groups of nurses and physicians as they work under different conditions. Hence, we hypothesize the following.

- Hypothesis 2: *Being a nurse or physician moderates the relationship between work experiences and organizational structures on an employee’s commitment to his or her organization.*

**Fig. 1:**
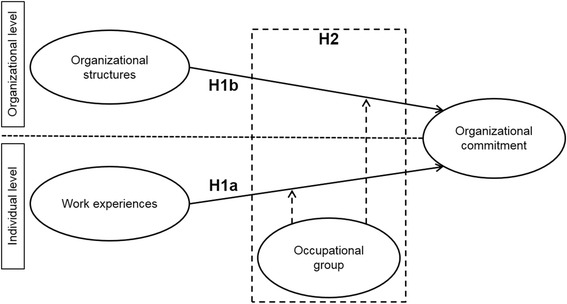
The hypothesized model

Figure 1 gives an overview of the research model.

## Methods

### Aim, design and setting of the study

The aim of the study was to examine the differential impact of individual and organizational factors on affective commitment for nurses and physicians to the organization.

As part of the Health Services Research in Neonatal Intensive Care (HSR-NICU) study, we conducted a cross-sectional multicenter study in which all existing German NICUs (*n* = 229) were asked to participate. In total, 66 NICUs (29%) agreed to take part in this study. Compared to all existing NICUs in Germany, participating hospitals were more publicly-owned (61% vs. 54%) and had, on average, more beds (*n* = 840 vs. *n* = 740). Nevertheless, the differences were not significant.

Irrespectively of their degree of qualification, all physicians and nurses working within respective NICUs were eligible to participate. 3045 staff questionnaires were administered to physicians and nurses in 66 participating NICUs. On average, 31 physicians and nurses per NICU participated in our study by completing a staff questionnaire, resulting in an overall response rate of 67.6% (*n* = 2059), including 1486 nurses and 540 physicians (33 participants did not provide information about their profession). The analysis was limited to employees for whom all included variables in the model were complete.

### Data collection

Preceding the actual staff questionnaire, all eligible nurses and physicians were provided with information on the overall content of the HSR-NICU research project and the subsequent procedure of the employee survey via a study brochure. Standardized postal questionnaires, which included questions about an employee’s work experiences and personal characteristics, as well as his or her commitment to the organization, were sent to NICUs in May 2013 afterwards. The principal investigator of each participating NICU was then asked to leave the questionnaires at a suitable location within the NICU to ensure that every nurse and physician had the possibility of participating, but was in no way obliged to participate. Completed questionnaires were anonymously returned in locked boxes 3 months later. To gather information about hospital sizes, the number of beds was retrieved from hospital quality reports. Information on the existence of regular interdisciplinary medical conferences was gathered from a self-administered structure survey completed by the medical director of each NICU. Data from these three different sources (employee questionnaires, hospital quality reports, self-administered structure surveys) were matched for the analysis.

### Measures

An overview of used survey items, including information about Likert scale range and descriptive statistics, is presented in Table 1.

**Table 1:** Scale properties and descriptive statistics

			Validity and reliability	
	Mean (SD) or n (%)^a^	Likert scale range^b^	No. of items	Cronbach’s α
Organizational Commitment	2.7 (0.7)	1 (*strongly disagree*) - 4 (*strongly agree*)	5	α = 0.86
Work experiences				
Support (from leader)	2.9 (0.7)	1 (*not at all*) - 4 (*completely*)	3	α = 0.87
Support (from colleagues)	3.2 (0.5)	1 (*not at all*) - 4 (*completely*)	3	α = 0.78
Autonomy in decision-making	3.4 (0.9)	1 (*strongly disagree*) - 5 (*strongly agree*)	3	α = 0.89
Autonomy in work methods	3.3 (0.8)	1 (*strongly disagree*) - 5 (*strongly agree*)	3	α = 0.84
Autonomy in work scheduling	3.4 (0.9)	1 (*strongly disagree*) - 5 (*strongly agree*)	3	α = 0.90
Organizational structures				Within-group agreement r_wg(j)_
Size (no. of hospital beds)	814 (582)			
Existence of regular interdisciplinary medical conferences (yes/no)	62 (3.13)			
Organizational support (from leader)	2.9 (0.3)	1 (*not at all*) - 4 (*completely*)	3	r_wg(j)_ = 0.76
Organizational support (from colleagues)	3.2 (0.1)	1 (*not at all*) - 4 (*completely*)	3	r_wg(j)_ = 0.88
Organizational autonomy in decision-making	3.4 (0.3)	1 (*strongly disagree*) - 5 (*strongly agree*)	3	r_wg(j)_ = 0.81
Organizational autonomy in work methods	3.3 (0.2)	1 (*strongly disagree*) - 5 (*strongly agree*)	3	r_wg(j)_ = 0.81
Organizational autonomy in work scheduling	3.4 (0.3)	1 (*strongly disagree*) - 5 (*strongly agree*)	3	r_wg(j)_ = 0.75
Open communication in organization	2.6 (0.2)	1 (*strongly disagree*) - 4 (*strongly agree*)	4	r_wg(j)_ = 0.86
Perceived quality of care in organization	3.1 (0.3)	1 (*strongly disagree*) - 4 (*strongly agree*)	3	r_wg(j)_ = 0.91
Control variables				
Executive position	203 (14.06)			
Gender (male)	191 (13.23)			
Age				
< 30	324 (22.44)			
30–50	954 (66.06)			
> 50	166 (11.50)			
Prof. experience in hospital^c^	17.3 (10.3)			
Prof. experience in respective unit^c^	10.9 (9.2)			
Employment status (full-time)	1011 (70.01)			

^a^Based on data in final model (*n* = 1444), percentages are presented with two digits, standard deviations (SD) with one digit; ^b^References for used scales are described in the “Measures” section of this manuscript; ^c^in years

To measure the affective commitment of an employee to his or her organization, we used the German validated version of affective commitment. The original scale showed good reliability according to Cronbach’s alpha (α = 0.86, [35]), and is based on the Affective Commitment Scale from Allen and Meyer [36].

To measure individual work experiences, support from leader and colleagues was assessed using an adaptation of the Caplan scale [37] by Udris and Riemann [38]. To investigate the multiple facets of autonomy, we used the Work Design Questionnaire (WDQ) from Morgeson and Humphrey [39] (German validation by Stegmann et al. [40]), which reflects different aspects of autonomy: The perceived freedom to make decisions (autonomy in decision-making) and to choose methods to perform tasks at work (autonomy in work methods), as well as the freedom to control the scheduling and timing (autonomy in work scheduling) of work. The original scales for support from leader and colleagues showed good reliabilities of α = 0.91 (leader) and α = 0.86 (colleagues) when utilizing it in the hospital setting [41]. Alpha reliabilities for autonomy ranged from .84 to .96 [40].

Organizational structures included the number of hospital beds (size) and the existence of regular interdisciplinary medical conferences*.* Open communication and perceived quality of care were derived from a questionnaire validated in the hospital setting [41].

In order to control for the potential influence of personal characteristics on the antecedent-outcome relationship, we controlled for staff characteristics that, based on previous literature [1], have recently been shown to be positively associated with organizational commitment: gender, age (0–30 years, 31–50 years, 51 years and older), working in an executive position (chief physician, senior physician, or head nurse), professional experience (years in hospital and respective unit), and employment status (full-time or not).

### Statistical analysis

To account for different levels of analysis (individual and organizational), we used hierarchical linear models with random intercepts and maximum-likelihood estimation to examine the hypotheses. In order to distinguish the individual-level effects from the unit-level effects, to avoid multicollinearity between different levels of analysis, and to facilitate the interpretation of the results, we centered the individual variables to the group mean and the unit-level variables (except for the dummy variables) to the grand mean [42]. Therefore, we estimated the following model:

$$ {Y}_{ij}={\beta}_{0j}+{\beta}_{10}\left({X}_{ij}-{\overline{X}}_j\right)+{\epsilon}_{ij} $$

with

$$ {\beta}_{0j}={\gamma}_{00}+{\beta}_{01}\left({Z}_j-\overline{Z}\right)+{u}_{0j} $$

under the assumption of *ϵ*_*ij*_ ∼ N(0,${\sigma}_{\epsilon}^2\Big)$and *u*_0*j*_ ∼ N(0,${\sigma}_u^2\Big),$where *Y*_*ij*_ is the perceived organizational commitment of staff *i* in unit *j*, *β*_0*j*_ is the random intercept as a function of the grand mean intercept *γ*_00_, the slope *β*_01_ for the unit grand-mean centered explanatory variables $\left({Z}_j-\overline{Z}\right)$ and a residual term *u*_0*j*_, *β*_10_ is the slope for the individual group-mean centered explanatory variables $\left({X}_{ij}-{\overline{X}}_j\right)$, and *ϵ*_*ij*_ is the residual term on the individual level [43]. Based on our research aim, we assumed fixed slopes within our models. As such, we did not take into account whether the strength of the relationship between the explanatory variables and commitment differed between units.

To conclude whether there were substantial differences in organizational commitment between organizations, a null model containing only the dependent variable was used to investigate the extent to which an employee’s organizational commitment could be explained by differences between units. Intra-class correlation (ICC) was used to calculate the between-group variance, i.e., the variance in organizational commitment that could be explained at the unit level [44].

Addressing Hypothesis 1a, we added individual work experiences and personal characteristics to test for significant effects on organizational commitment (Table 2, Model 1). Hypothesis 1b was tested by adding organization-level variables (Table 2, Model 2). We used the likelihood ratio to test whether the subsequent model was a significant improvement on the previous model. In the presence of model improvement, we calculated the between-group variance of the subsequent model and the percentage of between-group variance reduction, compared to the null model, to quantify how much of the variance could be explained by organizational-level variables.

**Table 2:** Individual and organizational antecedents of affective organizational commitment

	Affective organizational commitment^a^	
	Model 1	Model 2
Executive position	0.106^*^	0.107^*^
	(0.0520)	(0.0520)
Gender (male)	0.0192	0.0170
	(0.0525)	(0.0524)
Age (< 30)	−0.0433	− 0.0427
	(0.0497)	(0.0497)
Age (> 50)	0.169^**^	0.160^**^
	(0.0597)	(0.0597)
Professional experience in hospital (in years)	0.00225	0.00226
	(0.00316)	(0.00316)
Professional experience in respective unit (in years)	0.00591^*^	0.00602^*^
	(0.00288)	(0.00286)
Employment status (full-time)	0.0197	0.0217
	(0.0367)	(0.0366)
Support (from leader)	0.246^***^	0.245^***^
	(0.0240)	(0.0251)
Support (from colleagues)	0.115^***^	0.112^**^
	(0.0338)	(0.0343)
Autonomy in decision-making	0.0842^***^	0.0804^**^
	(0.0252)	(0.0256)
Autonomy in work methods	0.0325	0.0329
	(0.0262)	(0.0264)
Autonomy in work scheduling	0.0311	0.0271
	(0.0195)	(0.0198)
Size (No. of hospital beds)		−0.000122^**^
		(0.0000407)
Organizational support (from leader)		−0.124
		(0.102)
Organizational support (from colleagues)		−0.0663
		(0.203)
Organizational autonomy in decision-making		0.0236
		(0.150)
Organizational autonomy in work methods		0.0631
		(0.187)
Organizational autonomy in work scheduling		0.0822
		(0.108)
Open communication in organization		−0.108
		(0.164)
Perceived quality of care in organization		0.277^*^
		(0.113)
Existence of regular interdisciplinary medical conferences		0.289^*^
		(0.141)
Constant	2.545^***^	2.265^***^
	(0.0609)	(0.148)
Observations	1444	1444
ICC (null model: 9.90%)	8.07%	4.74%
Model improvement (likelihood-ratio test)	*p* < 0.001	*p* < 0.05

^a^Unstandardized beta coefficients, standard errors in parentheses; ^*^p < 0.05, ^**^p < 0.01, ^***^p < 0.001

To examine whether membership of an occupational group affected the relationship between the independent variables and an employee’s commitment (Hypothesis 2), moderating effects were tested by separately running random intercept models including personal characteristics as control variables, the interaction variable, and both main variables of the interaction variable for each independent variable from Model 2. Therefore, the occupational group (1 for a nurse and 0 for a physician) was added as a moderator of the individual-level relationship between an employee’s work experience and his or her organizational commitment and as a cross-level moderator to examine the effect of the occupational group in terms of organizational structures and commitment. Statistical analyses were performed using the statistical program Stata 11.2 (College Station, Texas, US).

### Validity, reliability, and rigor

The results of a pilot study formed the basis for utilizing the instruments within the setting of neonatal intensive care [20]. Based on the results of the pilot study, we adapted the constructs where necessary and again conducted a pretest in one NICU, which did not indicate any further changes. Confirmatory factor analysis was performed in the main study, where all items of the used constructs (with the exception of perceived quality of care, where one item was removed due to insufficient factor loading with an indicator reliability value less than 0.3) were retained. Reliability of the measures were tested using Cronbach’s alpha. Results indicate good to excellent reliability. Validity and reliability of used Likert-scales are presented in Table 1.

To measure organizational support from leader and colleagues, organizational autonomy, open communication, and the perceived quality of care within the organization, the average scores for these measures were calculated for each unit. To justify this aggregation from the individual to the unit level, within-group agreement (r_wg(j)_) was calculated according to James et al. [45]. As there has to be substantial homogeneity between staff members in terms of a specific construct, r_wg(j)_ measures the variability of a specific construct within a unit in relation to the variability expected by chance. According to James et al. [45], substantial within-unit agreement (> 0.7) justifies the aggregation of the construct at the unit level. Calculating r_wg(j)_ for each unit to justify aggregation at the unit level resulted, on average, in high levels of within-unit agreement. Results are reported in Table 1.

## Results

### Descriptive results

Overall, 1444 employees (1098 nurses and 346 physicians) from 64 NICUs where all included variables were complete (2 NICUs did not provide structural information) could be included. Table 1 shows the descriptive statistics for all participating employees.

Slightly more female (52.6%) than male physicians participated. Most of them worked full-time (92.2%), were older than 30 years (82.3%), and 35.3% worked in executive positions as senior or chief physicians. On average, physicians had 11.0 years of hospital experience (SD: 8.1) and 5.2 years of experience within the respective unit (SD: 5.7). In contrast, most of the nurses were female (97.5%), worked full-time (63.0%), were older than 30 years (76.1%), worked in non-executive positions (92.6%), and had longer hospital experience (Mean: 19.2, SD: 10.1) and experience within the respective unit (Mean: 12.6, SD: 9.3).

Participating employees reported, on average, high organizational commitment (Mean: 2.7, SD: 0.7), whereas physicians reported a slightly higher commitment (Mean: 2.8, SD: 0.7) to their hospital than nurses (Mean: 2.7, SD: 0.7).

### Individual and organizational antecedents of commitment

Overall, a significant proportion of variance in organizational commitment can be explained by differences between units (9.9%, *p* < 0.001). Nevertheless, the larger remaining part of variance in organizational commitment (90.1%) can be explained by differences between individuals.

Individuals with more years of professional experience (*p* < 0.05) in their unit, who were older than 50 (*p* < 0.01) and who held executive positions (*p* < 0.05), reported higher organizational commitment. As hypothesized (Hypothesis 1a), individual support from leader and colleagues and individual autonomy in decision-making were positively associated with organizational commitment (*p* < 0.001). There was no significant effect of individual autonomy in work methods and work scheduling on the commitment of nurses and physicians to the hospital.

Adding organizational predictor variables to the model, which included all individual-level variables, resulted in significant model improvement (*p* < 0.01) and a reduction in between-group variance of 41% (from 8.07% to 4.74%). Addressing Hypothesis 1b, we found a lower organizational commitment in larger hospitals (*p* < 0.01). In addition, in hospitals with a greater perceived quality of care, and where regular interdisciplinary medical conferences took place, higher employee commitment to the organization was reported (*p* < 0.05). Open communication, autonomy in the organization, and organizational support were not associated with an employee’s commitment. Table 2 gives an overview of the results.

### The moderating effect of occupational group

**Fig. 2:**
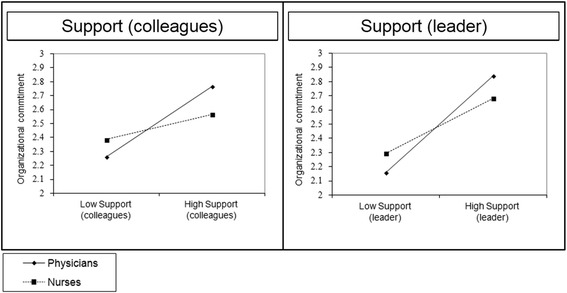
Individual support-commitment relationship by occupational group

Regarding work experience, we did not find significant differences in the influence of individual autonomy between both occupational groups. However, as shown in Fig. 2, the positive association between individual support from leader (*p* < 0.05) and colleagues (*p* < 0.001), and organizational commitment, was significantly stronger for individuals in the physician group.

There was no significant difference in the effect of hospital size, the existence of regular interdisciplinary medical conferences, open communication and organizational support from colleagues on commitment between the two occupational groups. However, in units with greater support from leader, greater autonomy in decision-making, work methods, and work scheduling, and a greater perceived quality of care, nurses reported significantly higher commitment than physicians. Significant results are shown in Table 3.

**Table 3:** Effect of organizational structures based on occupational groups^a^

	Affective organizational commitment^b^
Organizational support (from leader)	0.141
	(0.125)
Organizational support (from leader) X nurse	0.274*
	(0.121)
Organizational autonomy in decision-making	0.0657
	(0.137)
Organizational autonomy in decision-making X nurse	0.377**
	(0.132)
Organizational autonomy in work methods	0.00131
	(0.192)
Organizational autonomy in work methods X nurse	0.617***
	(0.187)
Organizational autonomy in work scheduling	0.117
	(0.150)
Organizational autonomy in work scheduling X nurse	0.355*
	(0.146)
Perceived quality of care in organization	0.0178
	(0.148)
Perceived quality of care in organization X nurse	0.591***
	(0.145)
Observations	1444

^a^Only those results are shown where significant differences in the effect of organizational structures on affective organizational commitment between occupational groups were found. All models include respective main effects and are adjusted for personal characteristics (executive position, gender, age, professional experience in hospital and respective unit, and employment status); ^b^ Unstandardized beta coefficients, standard errors in parentheses; *p < 0.05, **p < 0.01, ***p < 0.001

## Discussion

Using multilevel modeling allowed us to differentiate between the impact of predictor variables from different (individual and organizational) levels of analysis on individual affective commitment.

In particular, individuals who were older, had worked longer in their respective unit, or held an executive position reported greater commitment to their organization, an effect which was also shown in a meta-analysis across different settings [1]. Hence, this result might indicate a greater awareness of the needs and aspirations of healthcare professionals wherever intensive care units mainly consist of young and inexperienced staff.

Our finding of a positive association between perceived individual autonomy and support on employee commitment for both occupational groups (Hypothesis 1a) is consistent with the meta-analysis of Humphrey et al. [24] across different settings. However, the authors did not have enough studies to examine the association of different facets of autonomy and commitment. Nevertheless, they found a strong relationship of autonomy in decision-making and job satisfaction, which goes along with our findings, where only individual autonomy in decision-making was positively associated with organizational commitment. These associations reflect an important issue within the setting of acute and critical care: As physicians and nurses often face complex and unexpected situations in this setting, they require a work environment that is characterized by mutual support and the autonomy to make their own decisions. This can be fostered by management through the establishment of a participative leadership style and the promotion of nurse-physician collaboration [46], e.g., by establishing regular interdisciplinary meetings (an association that has been found to significantly influence an employee’s commitment in our study).

Several organizational structures were found to be associated with higher commitment (Hypothesis 1b). As Su et al. [26] found that larger organizations have a lower individual commitment, our results also indicate lower commitment of intensive care staff in hospitals with more beds. There are several explanations for this relationship. On the one hand, it might be more difficult for employees in larger hospitals to identify with their employer. Thus, larger hospitals in particular need to show greater effort to guard against low commitment by implementing structures designed to enhance an employee’s identification with his or her hospital, e.g., via hospital newsletters. On the other hand, employees in larger hospitals might perceive their relationships with their employers and co-workers to be weaker [26]. In our study, we found that interdisciplinary conferences as a regular occurrence were positively associated with an individual’s commitment. Hence, implementing such structures might be especially important for larger organizations to strengthen personal ties across professional boundaries. In addition, units with a strong emphasis on quality of care enhanced an employee’s commitment. As such, continuously working on the improvement of quality of care contributes to an employee’s outcome by enhancing his or her commitment to the organization.

In addition, our findings highlight the potential in and importance of differentiating between occupational groups.

The impact of individual support from leader and colleagues on commitment was stronger for physicians than for nurses. As a consequence, they might expect more individual support than nurses. However, these expectations are typically not met if a physician fails to voice demands. Considering the disparities in performed tasks between both occupational groups, it may be crucial that that physicians do not feel abandoned when confronted with difficult medical cases, as they are the ones who make the ultimate decisions regarding medical treatment. This is particularly important in so-called fast-response organizations, where fast decision-making is necessary and organizations are shaped by a high degree of uncertainty. This means that medical decisions are not always clear-cut and require a high degree of knowledge-sharing [47]. Physicians may require more individual support from their colleagues and leader to deal better with their responsibilities, which might also lead to more commitment to their organization. Hospital management should therefore enhance physician support, e.g., by establishing regular feedback mechanisms for physicians, which has also been previously shown to improve patient outcomes [48]. Our results also emphasize the particular need of physicians for supportive relationships with their supervisors and colleagues, an important issue that has been already emphasized by Galetta et al. [28], who showed the positive association between nurse-supervisor and nurse-physician relationships on nurses’ commitment.

In terms of the different effects of organizational structures for different occupational groups, nurse commitment was affected to a greater extent than physician commitment by a higher degree of organizational autonomy in decision-making, work scheduling, and work methods. This result highlights the importance of the ongoing debate in hospitals about the expansion of competencies for nurses within their profession [49] and underlines the problems of the nurses’ decision-making in critical care settings [50]. As nurses generally report a higher commitment to their organization in units where all professionals experience a high degree of autonomy, this could reflect the position of more equally shared competencies and the loosening of strict hierarchies between the two occupational groups. In addition, organizational support from leader had a stronger effect on commitment for nurses. In light of the results of Su et al. [26], which indicate a higher effect of organizational support only for employees at mid-level and lower-management levels, our results might reflect the greater need of a supportive environment in lower-level occupational groups.

Putting the study in a broader context, our findings contribute and are linked to the recent literature about the need to reform work arrangements and professional role types to enhance health care systems [51]. As our study emphasizes the need to concentrate on different strategies at the individual and organizational level, with a particular focus on the underlying occupational groups to increase individual commitment, the literature on engagement points out the need to develop strategies at the system level also [52]. In particular, the engagement of physicians in roles beyond their medical profession has been recently pointed out as an important managerial lever to improve performance in health care settings [53].

### Limitations and robustness of findings

Several limitations should be mentioned. First, because of our cross-sectional study design, we were not able to examine the causal link between our considered variables and an employee’s organizational commitment. For example, drawing upon existing research on the relationship between commitment and patient safety culture [16], one might also argue that enhancing the employee’s commitment might result in a better perceived quality. Second, the results where predictor and dependent variables were derived from the staff questionnaire could be biased because of common method variance. Therefore, we performed a split-sample strategy to exclude the possibility of common method variance in the organizational-level predictor variables on commitment [54]. The results showed that common method variance did not disturb our findings. To avoid the possible impact of common method variance on the individual-level predictor variables, we used proximal separation and different scale properties of the predictor and outcome variables [55]. Third, the within-group agreement of individual variables, which justifies aggregation at the unit level, could have been overestimated due to potential response bias. More specifically, in cases where the distribution of responses was non-uniform, i.e., participants were inclined to answer, for example, on the upper bound of alternatives in the response scale, we verified our results by recalculating the within-group coefficient r_wg(j)_ with the expected variance according to the underlying distribution [45]. After doing so, the results still showed sufficient within-group agreement for all organizational variables derived from the staff questionnaire. Fourth, as we used complete-case analysis where only data from employees with complete information on the variables of interest were analyzed, the results might be biased when missing values are not random. Although we cannot rule out this potential bias, we expect that missing data in our analysis did not depend on our dependent variable. As this might lead to negligible bias [56], we preferred this procedure to other possible techniques, i.e., multiple imputation. Finally, as this study was conducted in the environment of neonatal intensive care, we examined potential drivers of affective organizational commitment for a highly-educated and highly-specialized workforce. Although research on antecedents of commitment might be especially relevant for settings where professionals are even harder to replace due to their specialized skills, the extent to which our findings also apply to other hospital settings remains unclear.

## Conclusions

In accordance with findings of previous investigations [28], our study emphasizes the importance of examining factors driving an individual’s commitment, not only at the individual level, but also at unit level. Future studies should acknowledge the need to investigate drivers of commitment on different levels of analysis. In our study, however, we did not consider whether different factors might be associated with different aspects of affective commitment. The latest results of a study among nurses [57] emphasized the need to consider affective commitment in a more nuanced view. Hence, future studies might use, e.g., the Workplace Affective Commitment Multidimensional Questionnaire (WACMQ) [58], to investigate which targets of affective commitment might be influenced. In addition, this study extends our understanding of factors associated with organizational commitment with regard to the underlying occupational group. Our findings reveal that factors significantly associated with an individual’s organizational commitment differ between physicians and nurses. We recognize the urgent need to link the literature on the sociology of professions or the more focused literature on occupational differences in antecedent-commitment relationships to health care settings. In our study, we explicitly refrained from hypothesizing which factors might be more important for which occupational group, due to the lack of theoretical foundations. Further research is required to connect our conceptual model more closely to theoretical development.

In conclusion, our results indicate that more specific management actions corresponding to the different needs of physicians and nurses could be undertaken in order to improve employee commitment and, in the long run, reduce turnover intentions and improve patient safety [16]. This is particularly important in intensive healthcare settings with critically ill patients and highly specialized professionals, who are difficult – and often costly – to replace.

## Abbreviations

HSR-NICU: Health Services Research in Neonatal Intensive Care
ICC: Intra-class correlation
NICU: Neonatal Intensive Care Unit
r_wg(j)_: Within-group agreement
